# Association of Media Coverage on Transgender Health With Referrals to Child and Adolescent Gender Identity Clinics in Sweden

**DOI:** 10.1001/jamanetworkopen.2021.46531

**Published:** 2022-02-02

**Authors:** Malin Indremo, Anna Clara Jodensvi, Hans Arinell, Johan Isaksson, Fotios C. Papadopoulos

**Affiliations:** 1 Department of Neuroscience, Psychiatry, Uppsala University, Uppsala, Sweden; 2 Center of Neurodevelopmental Disorders, Centre for Psychiatry Research, Department of Women’s and Children’s Health, Karolinska Institute, Stockholm, Sweden

## Abstract

**Importance:**

Increasing numbers of transgender and gender diverse children and adolescents have been referred to gender identity clinics in Sweden in the past decade, and previous studies have found an association between media attention and referral counts. Whether the tone of media stories is associated with referrals is not yet known.

**Objective:**

To investigate whether positive or negative media coverage on transgender and gender diverse issues is associated with referral counts to child and adolescent gender identity clinics.

**Design, Setting, and Participants:**

In this cross-sectional study, referrals counts were collected from all 6 specialized gender identity clinics in Sweden, along with information on referral date, sex assigned at birth, and birth year. Participants were all individuals younger than 19 years, referred to a gender identity clinic between January 1, 2017, and December 31, 2019. Data were analyzed from May 2020 to September 2021.

**Exposures:**

Exposures included 3 major media events related to transgender health care in 2019; 1 event was categorized as positive and 2 events were categorized as negative press coverage.

**Main Outcomes and Measures:**

Referral counts before and after each of the 3 major media events were assessed, and the moderating association of sex assigned at birth and age was examined with stratified analyses. Weekly referral counts and trends were assessed with interrupted time series analysis.

**Results:**

Among 1784 referrals (359 referrals [21.4%] among individuals aged <13 years; 1034 referrals [72.1%] among individuals assigned female at birth and 401 referrals [27.9%] among individuals assigned male at birth), a negative association between media coverage and referral counts was found for the first of the negative media events. In the 3 months following the event, referrals decreased by 25.4% (95% CI, −31.9% to −18.9%) overall, by 32.2% (95 CI, −41.8% to −22.8%) for individuals assigned female at birth, and by 25.3% (95 CI, −32.4% to −18.3%) for individuals aged 13 to 18 years. In the interrupted time series analysis, a mean weekly decrease of 3% in the referrals was observed in the extended time interval of 39 weeks (incidence rate ratio, 0.97; 95 CI, 0.95 to 0.99). For the other 2 media events, no changes in referral counts or time trends were observed.

**Conclusions and Relevance:**

These findings suggest that an event of negative media coverage on transgender-specific health care was negatively associated with access to health care for transgender children and adolescents; therefore, nuanced and accurate media coverage, as well as increased awareness of these mechanisms among key stakeholders, is essential.

## Key Points

**Question:**

Are positive and negative media coverage of transgender health care associated with referrals to child and adolescent gender identity clinics in Sweden?

**Findings:**

In this cross-sectional study including 1784 referrals, negative media coverage on transgender health care was associated with decreased referrals to gender identity clinics. Referrals decreased by 25.2% over a 3-month period following 1 critical media event.

**Meaning:**

These findings suggest that media coverage may influence and jeopardize young transgender individuals’ access to transgender-specific health care; therefore, nuanced and accurate media coverage is essential.

## Teaser

To investigate whether the tone of media coverage on transgender and gender diverse issues is associated with referral counts to child and adolescent gender identity clinics in Sweden.

## Introduction

In recent years, the number of referrals to gender identity clinics have rapidly increased worldwide.^1,2^ Gender identity clinics offer assessments and possible treatments for gender dysphoria (GD), which is characterized by the *Diagnostic and Statistical Manual of Mental Disorders* (Fifth Edition)^3^ as significant suffering or impairment in functioning due to incongruence between the individual’s experienced gender and their birth-assigned sex. In children, this is often accentuated by the onset of puberty and development of secondary sex characteristics.^4^ Accordingly, the increase of referrals to gender identity clinics has been especially prominent in adolescents and young adults.^5,6,7^ In addition, there has been a shift in gender ratio, with a preponderance toward individuals who were assigned female at birth (AFAB).^8,9,10^ Whether the increase reflects a change in the actual prevalence of GD in the population, an increase in help-seeking behavior, or a combination of both is not yet known. Several explanations that could influence care-seeking behavior among individuals with GD have been proposed, including enhanced availability of information on gender issues on the internet, decreased stigmatization and increased acceptance in society, and more accessible health care, as well as increased attention of transgender issues in media.^2,5,11^

Several studies have highlighted the importance of increased visibility of transgender individuals in the media,^12,13,14^ and exposure to storylines depicting transgender individuals is associated with more supportive attitudes toward transgender people and policies.^15^ Corroborating this, results from qualitative studies based on interviews with young transgender individuals indicate that positive news stories provide a sense of hope and resilience, whereas negative news stories are associated with increased feelings of depression and anxiety, resulting in decreased mental well-being.^13,14,15^ Studies on possible associations between media coverage and care-seeking behavior in individuals with GD are scarce. To our knowledge, there is only 1 study examining the association between referral rates to gender identity clinics and transgender-related media coverage. Pang and colleagues^16^ conducted a cross-sectional study in the UK and Australia and concluded that the number of media events focusing on transgender and gender diverse issues was associated with increased referral rates 1 to 2 weeks after the media coverage. However, the study by Pang et al^16^ did not distinguish between positive and negative media attention, and the authors emphasized the need to further address if negative press coverage may be associated with reduced referral rates.

To further examine the role of media in care-seeking behavior, the primary aim of this study was to examine the association between negative and positive media coverage and the national inflow of referrals to gender identity clinics evaluating individuals younger than 19 years in Sweden. Since 1 of the negative media events focused on teenaged AFAB individuals, the secondary aim was to analyze if this possible association would be moderated by sex assigned at birth and age. Based on earlier evidence that media attention may influence a sense of resilience in transgender individuals, our hypothesis was that positive media coverage would be positively associated with referral counts, whereas negative media coverage would be negatively associated with the number of incoming referrals.

## Methods

This cross-sectional study included only anonymized data that cannot be traced back to any living individual; therefore, it was exempt from human participants committee approval. In accordance with Swedish ethics review law,^24^ the Swedish ethics committee does not grant waivers on studies that do not contain any personal data, nor is informed consent required.^25^ The study followed the Strengthening the Reporting of Observational Studies in Epidemiology (STROBE) reporting guideline.

### Media Coverage

A search on the Swedish term for gender dysphoria (*könsdysfori*) on Google trends^17^ the past 3 years demonstrated distinct peaks in search interest in January 2019, in the beginning of April 2019, and a smaller increase in the beginning of October 2019. These peaks coincide with 3 different media events, referred to as events 1, 2, and 3. On January 7, 2019, a professional Swedish handball player announced the decision to quit his career to be able to seek care for GD that he had been experiencing for a long time.^18^ The announcement (event 1) was given extensive positive media coverage and will be considered as positive media coverage in this analysis. The 2 latter peaks coincide with two documentaries called “The Trans Train and the Teenage Girls,”^19^ a 2-part documentary series broadcast on April 3, 2019 (event 2), and October 9, 2019 (event 3), by a Swedish public service television show for investigative journalism. The documentaries addressed the distinct increase among adolescents referred to gender identity clinics in recent years. Two young adults who regretted their transition and parents of transgender individuals who questioned the clinics’ assessments of their children were interviewed, and concerns were raised about whether gender confirming treatments are based on sufficient scientific evidence. An intense debate in national media arose from the documentaries,^20,21^ and the documentaries were criticized for being negatively biased and giving an oversimplified picture of transgender health care.^22,23^ These events will be considered as negative media coverage for this analysis.

### Study Design and Data Collection

A cross-sectional design was used to examine the association between 3 media events and referral counts to gender identity clinics for individuals younger than 19 years in Sweden between January 1, 2017, and December 31, 2019. There are 6 gender identity clinics in Sweden, which were all contacted and asked to share the number of referrals to their clinic for all individuals younger than 19 years, along with information specified for each referral, including referral date, birth year, and sex assigned at birth. In some regions, adolescents older than 16 years are referred directly to the adult clinics; therefore, the corresponding clinics were contacted and asked to provide supplementary information.

Owing to organizational disparities among clinics in how referral counts are registered, characteristics in provided data differed. Three clinics provided exact referral dates for all referrals and 1 clinic provided information on referral week. These data were aggregated into monthly and weekly referral counts and stratified by sex assigned at birth and age group (<13 years vs 13-18 years). Two clinics reported only referral month and hence were excluded from the weekly analyses (394 referrals). One binary variable for each of the 3 media events and for each time window was created and added to the data sets indicating if the observed referrals were in the pre-event or postevent period. Information on sex assigned at birth was lacking in the data from 1 clinic (349 referrals) and hence data from that clinic were excluded from the sex-specific analyses. Data on age group were lacking from another clinic (110 referrals), and that clinic was excluded from the age-specific analyses. Details on data characteristics provided by the clinics, as well as descriptions on data processing, are presented in eTable 1 in the Supplement.

### Statistical Analysis

Initial descriptive statistics on monthly referral counts were undertaken for all referrals and stratified by assigned sex and age category. We calculated the differences in monthly referral counts during a 3-month period preceding and following the media events. This time frame was chosen to avoid having overlapping periods between the events. For a media event with a significant alteration in referral counts, the time interval was extended from 3 to 6 months to evaluate if an association could be observed during a prolonged time window.

An interrupted time series regression analysis was used to compare weekly referral rates and time trends before and after the media events. Negative binomial distribution was chosen since it had better model fit compared with Poisson distribution. In model 1, incidence rate ratios (IRRs), along with their 95% CIs, were assessed for the weekly referrals after the event compared with the referrals before the event. In model 2, we included the interaction term between event and time, and in model 3, we included the interaction term of event and time as well as a dummy variable for seasonality, to adjust for any seasonal confounding. The interrupted time series analysis provides independent tests of the underlying trend in referrals before and after the events, as well as the level change in referrals the weeks before and after the event. Time series of 12 weeks before and after for each of the 3 media events were analyzed with both model 1 and 2. Analyses of 39 weeks before and after the event were performed for 1 event found to have significant results in the 12-week analyses, including correction for seasonality (model 3). The time period of 39 weeks was selected because that was the maximum number of weeks with available data past the event achieving equal number of data points before the event.

We controlled for autocorrelation between observations with a 1- to 16-week lag. Analysis showed no autocorrelation lag (>0.3), and therefore no autocorrelation term was included in the models. Data were analyzed in SPSS Statistics version 26.0 (IBM). *P* values were 2-sided, and statistical significance was set at *P* = .05. Our first analyses were conducted from May 7 to May 23, 2020, and additional analyses were conducted from September 6 to September 14, 2021.

## Results

### Referral Counts

**Figure 1.**
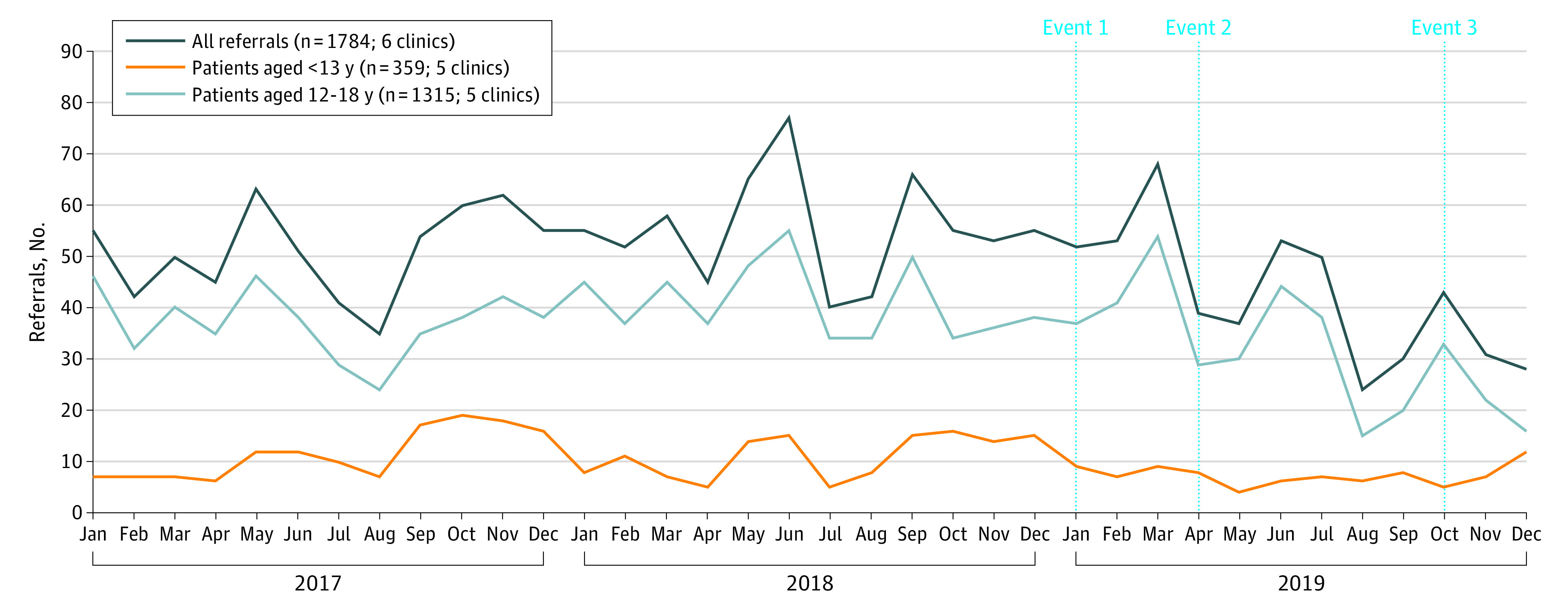
Referrals to Gender Identity Clinics From 2017 to 2019 in Total and by Age

**Figure 2.**
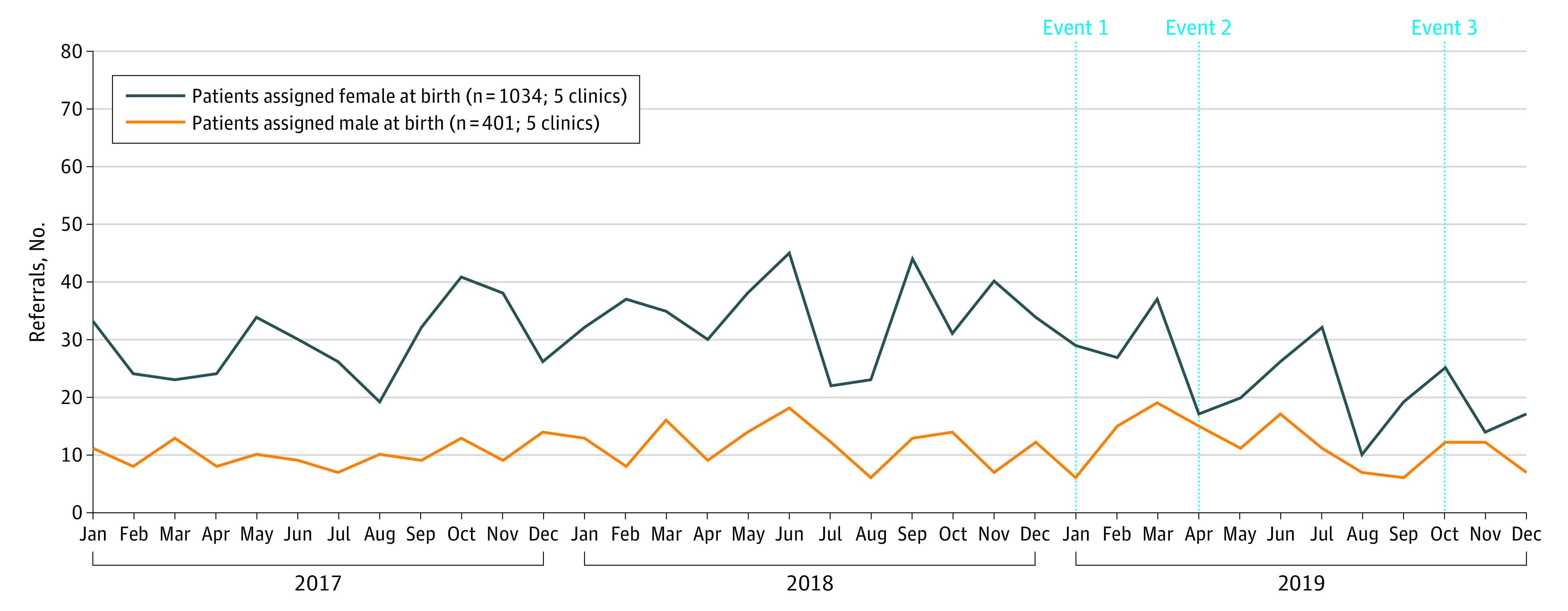
Referrals to Specialist Gender Identity Clinics From 2017 to 2019, Grouped by Sex Assigned at Birth

The gender identity clinics received 1784 referrals during the study period, including 613 referrals in 2017, 663 referrals in 2018, and 508 referrals in 2019. From the age-specific data including 1674 referrals, 359 individuals (21.4%) were younger than 13 years and 1315 individuals (78.6%) were aged 13 to 18 years. From the assigned sex–specific data including 1435 referrals, 1034 individuals (72.1%) were AFAB and 401 individuals (27.9%) were assigned male at birth (AMAB). Monthly referrals in total and by age are presented in Figure 1, and by assigned sex in Figure 2. Data on weekly referral counts for 1390 referrals, provided by 4 clinics, varied between 0 to 20 referrals, as displayed in the eFigure in the Supplement.

### Event 1: Positive Media Coverage

Clinics received 163 referrals in the 3-month period preceding event 1 and 173 referrals after the event. No significant changes were observed before vs after the event, either when analyzed by all referrals or when stratified by age or assigned sex (Table 1). Corresponding calculations for the whole time period 2017 to 2019 are presented in eTable 2 in the Supplement.

**Table 1.** Referrals During 3 Months Before and After Media Event 1 and Media Event 3

Category	Referrals before the event	Referrals after the event	% Change (95% CI)
**Event 1 **			
All	163	173	6.1 (2.2 to 10.1)
Sex assigned at birth			
Female	105	93	−11.4 (−17.5 to −5.3)
Male	33	40	21.2 (3.9 to 38.5)
Age, y			
<13 y	45	27	−40.0 (−54.3 to −25.7)
13-18 y	118	146	23.7 (14.0 to 33.5)
**Event 3 **			
All	104	102	−1.9 (−4.6 to 0.7)
Sex assigned at birth			
Female	61	56	−8.2 (−15.1 to −1.3)
Male	24	31	29.2 (4.6 to 53.7)
Age, y			
<13	22	25	13.6 (−2.8 to 30.1)
13-18	82	77	−6.1 (−11.3 to −0.9)

### Event 2: Negative Media Coverage

Clinics received 173 referrals in the 3-month period preceding event 2 and 129 referrals after the event. In the 3 months following event 2, the total number of referrals decreased by 25.4% (95% CI, −31.9% to −18.9%), by 32.2% (95% CI, −41.8% to −22.8%) for AFAB individuals, and by 25.3% (95% CI, −32.4% to −18.3%) in individuals aged 13 to 18 years, compared with the respective previous 3-month period.

In the extended analyses of 6 months, a decrease of total referrals by 30.7% (95% CI, −35.6% to −25.7%) was observed, while the referrals for AFAB individuals decreased by 37.4% (95% CI, −44.1% to −30.9%), and referrals for individuals aged 13 to 18 years decreased by 27.7% (95% CI, −33.0% to −22.3%). A decrease of referrals by 41.7% (95% CI, −53%-1% to −30.3%) for children aged younger than 13 years was observed only in the 6-month analysis, as well as a decrease of 8.2% (95% CI, −14.5 to −1.9) among AMAB individuals (Table 2).

**Table 2.** Referrals During 3 and 6 Months Before and After Media Event 2

Category	3 months			6 months		
	Referrals before event, No.	Referrals after event, No.	% Change (95% CI)	Referrals before event, No.	Referrals after event, No.	% Change (95% CI)
All	173	129	−25.4 (−31.9 to −18.9)	336	233	−30.7 (−35.6 to −25.7)
Sex assigned at birth						
Female	93	63	−32.3 (−41.8 to −22.8)	198	124	−37.4 (−44.1 to −30.6)
Male	40	43	7.5 (−1.3 to 16.3)	73	67	−8.2 (−14.5 to −1.9)
Age, y						
<13	27	20	−25.9 (−42.5 to −9.4)	72	42	−41.7 (−53.1 to −30.3)
13-18	146	109	−25.3 (−32.4 to −18.3)	264	191	−27.7 (−33.0 to −22.3)

### Event 3: Negative Media Coverage

Clinics received 104 referrals in the 3 months preceding event 3 and 102 referrals after the event. No significant changes were observed before vs after the event, either when analyzed by all referrals or when stratified by age or sex assigned at birth (Table 1).

### Interrupted Time Series Analyses

The interrupted time series analyses showed no significant association of event 1 (IRR, 1.13; 95% CI, 0.80 to 1.57) or of event 3 (IRR, 1.12; 95% CI, 0.79 to 1.58) with referrals (Table 3). Regarding event 2, the mean referral counts decreased by 30% (IRR, 0.70; 95% CI, 0.53 to 0.94) in the 12 weeks after event 2 and by 31% (IRR, 0.69; 95% CI, 0.56 to 0.86) in the 39 weeks after. Analyzing the weekly trend, a significant time trend with a weekly decrease of 3% was observed after the event in model 3, adjusting for seasonality in the 39-week time window (IRR, 0.97; 95% CI, 0.95 to 0.99). Using model 2 that included only the interaction term of time and a shorter time window of 12 weeks, no statistically significant difference was observed (IRR, 0.96; 95% CI, 0.88 to 1.04). No significant level changes were observed for any of the events or models.

**Table 3.** Negative Binomial Regression Models for Associations Between the Media Events and Referral Counts

Event	Period, wk	Model 1^a^			Model 2 (ITS analysis)^b^			Model 3 (ITS analysis adjusted for seasonality)^c^		
		Mean weekly referrals, No.		IRR (95% CI)^d^	Trend (95% CI)		Level change (95% CI)	Trend (95% CI)		Level change (95% CI)
		Pre-event	Postevent		Pre-event	Postevent		Pre-event	Postevent	
1	12	8.92	10.08	1.13 (0.80 to 1.57)	0.99 (0.92 to 1.07)	1.04 (0.94 to 1.15)	−0.02 (−5.91 to 5.89)	NA	NA	NA
2	12	10.08	7.08	0.70 (0.53 to 0.94)	1.03 (0.98 to 1.09)	0.96 (0.88 to 1.04)	−4.22 (−9.47 to 1.02)	NA	NA	NA
	39	9.41	6.48	0.69 (0.56 to 0.86)	1.01 (0.99 to 1.02)	0.98 (0.96 to 1.11)	−2.39 (−6.23 to 1.46)	1.01 (0.99 to 1.03)	0.97 (0.95 to 0.99)	−2.80 (−8.23 to 2.66)
3	12	5.08	5.67	1.12 (0.79 to 1.58)	1.03 (0.95 to 1.10)	0.96 (0.87 to 1.06)	0.37 (−3.71 to 4.46)	NA	NA	NA

Abbreviation: ITS, interrupted time series analysis; IRR, incidence rate ratio; NA, not applicable.

^a^ Weekly referrals were the outcome measure in all models. The explanatory variable in model 1 was only the event variable.

^b^ The explanatory variable in model 2 was the event, the time, and the interaction term event × time.

^c^ The explanatory variable in model 3 constituted of model 2 with the addition of the seasonality dummy variable.

^d^ IRRs are given for weekly referrals before vs after the event.

## Discussion

In this cross-sectional study, we investigated the association between negative and positive media coverage and the national inflow of referrals to specialized gender identity clinics in Sweden for children and adolescents during 2017 to 2019. In line with one of our hypotheses, a negative association between media attention and referral counts was found for the first of 2 negative media events; referrals regarding AFAB individuals and in individuals aged 13 to 18 years decreased during 3- and 6-month periods after the event. In the weekly analyses, a decrease of mean referral counts per week after the event was observed up to 39 weeks after the negative media event. In contrast to our hypothesis, no alterations in referrals were seen associated with the positive media coverage event.

Previous research has reported associations between media coverage and attitudes as well as care-seeking behavior across different settings, including suicide prevention, cancer-related services, and transgender and gender diverse issues.^12,14,15,26,27,28,29^ In the only study on the association between referral counts to gender identity clinics and transgender -related media coverage, to our knowledge, Pang and colleagues^16^ reported that the number of media events was associated with increased referral counts.

Our results point to a differential association of media attention depending on the tone of the media content. We focused on 3 media events given extensive coverage in Sweden during a limited time period, whereas Pang et al^16^ analyzed a large of number of media items, also including items of lower impact, during a longer period and weighing each media item equally.

Regarding the media event given positive coverage in our study, for which no association was found with referrals, one may assume that a single news event is not significant enough to influence referral counts. Moreover, there might be a ceiling effect in which a single positive event has no substantial association in Sweden, a society where there is already a relatively high level of awareness of gender identity issues.^30^ In comparison, the negative media events (events 2 and 3) were given extensive media attention for prolonged periods after being broadcast. The fact that a decrease in referral counts was not observed after event 3 could be associated with the referral counts being already lowered in the time of the second documentary, possibly an association of the first documentary (event 2). It is also critical to point out that the decrease of referrals was mostly observed in the decrease of referred AFAB individuals aged 13 to 18 years, the group in focus in event 2. Our results do not provide evidence of causation; however, the findings are consistent with the clinical experiences from gender identity clinics that the documentaries and the following debate resulted in several patients’ experiences of increased difficulties getting referred to a gender identity clinic.

In a study on experienced barriers to gender-affirming health care, Pucket and colleagues^31^ identified several obstacles for pursuing gender-affirming health care. The need of parental consent among minors, bias among medical and mental health personnel, poor availability of care, worries about treatment, and interpersonal barriers were among the reported obstacles. It is possible that the content in the documentaries (events 2 and 3) may have contributed to such barriers.

First, the first documentary (event 2) in particular examined the perspectives of worried parents who questioned that their children were experiencing GD and worried that the children may be prescribed irreversible medical treatments they may later regret. For children and adolescents to access gender-confirming health care in Sweden, they need both custodians’ permission as well as their custodians’ help facilitating the contact with the specialized clinics. It is possible that the content of the documentary (event 2) contributed to a higher custodian barrier to having their children referred for assessment, believing it may not be in the best interest of their child. This would highly impact young transgender individual’s possibilities to access care.

Second, the attitudes of health care practitioners referring children for evaluation of GD might also have been influenced by the documentary. Most of the gender identity clinics in Sweden require referrals from other health care practitioners, who may have been more reluctant to refer children and adolescents after seeing the documentaries. After the documentaries, some commenters argued that all gender-confirming treatments for adolescents and young adults should be stopped^32^ and that all health care given at the gender identity clinics was an experiment lacking scientific basis.^20^

Third, negative media portrayal of transgender issues has in previous studies been identified as associated with influencing people’s attitudes toward transgender individuals and policies.^13^ One could also argue that the documentaries highlighted actual shortcomings in the assessment protocols of the clinics, and in accordance, the demand for their services decreased. However, the documentary received criticism for presenting a biased and 1-sided picture of transgender health care, with descriptions of the protocols being partly erroneous.^22^ With this in mind, it is possible that the documentary may have increased barriers of stigma and lack of social acceptance, previously identified as obstacles to seek gender-affirming health care.

### Limitations

This study has some limitations. The collected data on referrals were heterogenous owing to differences in the administrative workflows in each clinic. However, since the focus in this study was to analyze the changes in association with the media events, rather than the absolute numbers of referrals, the risk of bias is considered to be minimal. Information on sex assigned at birth was lacking in the data from 1 clinic, and data from that clinic were excluded from the sex assigned at birth–specific analyses. Similarly, there was some heterogeneity regarding whether the data included both accepted and refused referrals; all centers but 1 provided data on both. However, each clinic reported their referrals homogenously over the whole 3-year period, making comparisons among years possible. Moreover, there may have been differences in reach and accessibility among the 3 media events. To increase the understanding of these mechanisms, future research should explore the reactions to relevant media events by the different parties involved in referring a child or adolescent to a gender identity clinic, ie, the child or adolescent, the parents, and the referring health care professionals. Furthermore, we cannot draw conclusions regarding any media event based on the results from our study, but our results may indicate an association between referral rates and high-impact negative media coverage. To determine whether these results would be applicable in a different cultural context, replication studies in other countries would be necessary.

## Conclusions

In this nationwide cross-sectional study, we found an association between an event of negative media coverage on transgender health care with decreased referral counts to gender identity clinics in Sweden. Given that GD is associated with substantial mental and physical health burden, nuanced and accurate media coverage, as well as increased awareness of these mechanisms among key stakeholders, are essential.

## Notes

Supplement. **eTable 1.** Characteristics of the Data Provided by the Clinics and Processing, and Analyses Information **eTable 2.** Referral Counts and Percentage Changes in 3 Months Before and After the Media Events During 2017 to 2019 **eFigure.** Weekly Referrals to Gender Identity Clinics During 2017 to 2019
