# Supplementary material for: Association of Media Coverage on Transgender Health With Referrals to Child and Adolescent Gender Identity Clinics in Sweden
Source: JAMA Netw Open. 2022 Feb 2;5(2):e2146531. doi: 10.1001/jamanetworkopen.2021.46531 (PMC8811638; doi:10.1001/jamanetworkopen.2021.46531)
Supplement: Supplement. — eTable 1. Characteristics of the Data Provided by the Clinics and Processing, and Analyses Information eTable 2. Referral Counts and Percentage Changes in 3 Months Before and After the Media Events During 2017 to 2019 eFigure. Weekly Referrals to Gender Identity Clinics During 2017 to 2019 [file jamanetwopen-e2146531-s001.pdf]

Supplemental Online Content

Indremo M, Jodensvi AC, Arinell H, Isaksson J, Papadopoulos FC. Association of media coverage on transgender health with referrals to child and adolescent gender identity clinics in Sweden. *JAMA Netw Open*. 2022;5(2):e2146531. doi:10.1001/jamanetworkopen.2021.46531

eTable 1. Characteristics of the Data Provided by the Clinics and Processing, and Analyses Information
eTable 2. Referral Counts and Percentage Changes in 3 Months Before and After the Media Events During 2017 to 2019
eFigure. Weekly Referrals to Gender Identity Clinics During 2017 to 2019

This supplemental material has been provided by the authors to give readers additional information about their work.

© 2022 Indremo M et al. *JAMA Network Open.*

eTable 1. Characteristics of the Data Provided by the Clinics and Processing, and Analyses Information

Clinic and age restrictions	Data characteristics	Data processing and analyses	Comments
**Alingsås (child and adolescent clinic)**<br><br><18 years	Information on referral date was provided.<br><br>Both accepted and refused referrals.<br><br>No information on assigned sex provided.	Analyzed in both weekly and monthly analyses.<br><br>Both accepted and refused referrals included in analyses.<br><br>The referrals were excluded from the sex-specific analyses.	-
**Stockholm (child and adolescent clinic)**<br><br><18 years	Information on referral week (isoweek) was provided. No information on referral month or date.<br><br>Accepted referrals only.<br><br>No information on birth year was provided. Referrals were categorized into two age groups: ≤12 years and 13-18 years.	Analyzed in both weekly and monthly analyses.<br><br>Referrals were grouped by month based on week number.	-
**Umeå (child and adolescent clinic)**<br><br><18 years	Information on referral date was provided.<br><br>Both accepted and refused referrals.	Analyzed in both weekly and monthly analyses.<br><br>Both accepted and refused referrals included in analyses.	-
**Lund (child and adolescent clinic)**<br><br><18 years	Information on referral date was provided.<br><br>Both accepted and refused referrals.	Analyzed in both weekly and monthly analyses.<br><br>Both accepted and refused referrals included in analyses.	Clinic opened in 2018. The child and adolescent clinic and the adult clinic were the same in 2017.
**Lund (adult clinic)**<br><br><18 years starting in January-March 2017. Before the lower age limit for referral here was >16 years.	Information on referral date was provided.<br><br>Both accepted and refused referrals.	Analyzed in both weekly and monthly analyses.<br><br>Both accepted and refused referrals included in analyses.	Referrals from 2017.<br><br>The lower age restriction of 16 years was removed and replaced with no lower age restriction in January-March 2017. The change was implemented gradually, therefore only a few of the referrals (n=4) were <16 years.
**Uppsala (child and adolescent clinic)**<br><br><16 years	Information on referral month was provided.	Analyzed in monthly analyses only.	-

© 2022 Indremo M et al. *JAMA Network Open.*

	Both accepted and refused referrals.	Both accepted and refused referrals included in analyses.	
**Uppsala (adult clinic)**<br><br>16-18 years	Information on referral date was provided.<br><br>Accepted referrals only.	Analyzed in monthly analyses only (since the children and adolescent clinic included only monthly data).	-
**Linköping (child and adolescent clinic)**<br><br><18 years	Information on referral date was provided.<br><br>Both accepted and refused referrals.	Analyzed in monthly analyses only (since the corresponding adult clinic included only monthly data).<br><br>The referrals forwarded to the adult clinic were excluded.<br><br>Both accepted and refused referrals included in analyses.	The clinic had a referral stop in 2018 due to lack of personnel. Still n=7 referrals was registered in 2018. Patients were referred to the clinic in Stockholm during this time period.
**Linköping (adult clinic)**<br><br>17-18 years	Information on referral month was provided.<br><br>Both accepted and refused referrals.	Analyzed in monthly analyses only.<br><br>Both accepted and refused referrals included in analyses.	The clinic has an agreement with the child and adolescent clinic in Linköping to take over referrals aged 17-18 years.

© 2022 Indremo M et al. *JAMA Network Open.*

eTable 2. Referral Counts and Percentage Changes in 3 Months Before and After the Media Events During 2017 to 2019

EVENT 1	2017 – 2018				2018 - 2019							
	Oct-Dec	Jan-Mar	% Change	95% CI	Oct-Dec	Jan-Mar	% Change	95% CI				
All	177	165	-6.8	-10.5 to -3.1	163	173	6.1	2.2 to 10.1				
Assigned females	105	104	-1.0	-2.8 to 0.9	105	93	-11.4	-17.5 to -5.3				
Assigned males	36	37	2.8	-2.7 to 8.3	33	40	21.2	3.9 to 38.5				
<13 years	53	26	-50.9	-64.4 to -37.5	45	27	-40.0	-54.3 to-25.7				
13-18 years	124	139	12.1	5.6 to 18 .6	118	146	23.7	14.0 to 33.5				
EVENT 2	2017				2018				2019			
	Jan-Mar	Apr-Jun	% Change	95% CI	Jan-Mar	Apr-Jun	% Change	95% CI	Jan-Mar	Apr-Jun	% Change	95% CI
All	147	159	8.2	3.4 to 13.0	165	187	13.3	7.4 to 19.3	173	129	-25.4	-31.9 to -18.9
Assigned females	80	88	10.0	2.7 to 17.3	104	113	8.7	2.8 to 14.5	93	63	-32.3	-41.8 to -22.8
Assigned males	32	27	-15.6	-28.2 to -3.0	37	41	10.8	-0.3 to 22.0	40	43	7.5	-1.3 to 16.3
<13 years	22	31	40.9	9.2 to 72.6	26	35	34.6	8.4 to 60.9	27	20	-25.9	-42.5 to -9.4
13-18 years	125	128	2.4	-0.3 to 5.1	139	152	9.4	4.0 to 14.7	146	109	-25.3	-32.4 to 18.3
EVENT 3	2017				2018				2019			
	Jun-Sep	Oct-Dec	% Change	95% CI	Jun-Sep	Oct-Dec	% Change	95% CI	Jun-Sep	Oct-Dec	% Change	95% CI
All	130	177	36.2	24.1 to 48.2	148	163	10.1	4.8 to 15.5	104	102	-1.9	-4.6 to 0.7
Assigned females	77	105	36.4	20.6 to 52.1	89	105	18.0	8.4 to 27.5	61	56	-8.2	-15.1 to -1.3
Assigned males	26	36	38.5	10.4 to 66.5	31	33	6.5	-2.8 to 15.7	24	31	29.2	4.6 to 53.7
<13 years	34	53	55.9	24.5 to 87.3	29	45	55.2	21.5 to 88.8	22	25	13.6	-2.8 to 30.1
13-18 years	96	124	29.2	16.9 to 41.4	119	118	-0.8	-2.5 to 0.8	82	77	-6.1	-11.3 to -0.9

© 2022 Indremo M et al. *JAMA Network Open.*

eFigure. Weekly Referrals to Gender Identity Clinics During 2017 to 2019[a]

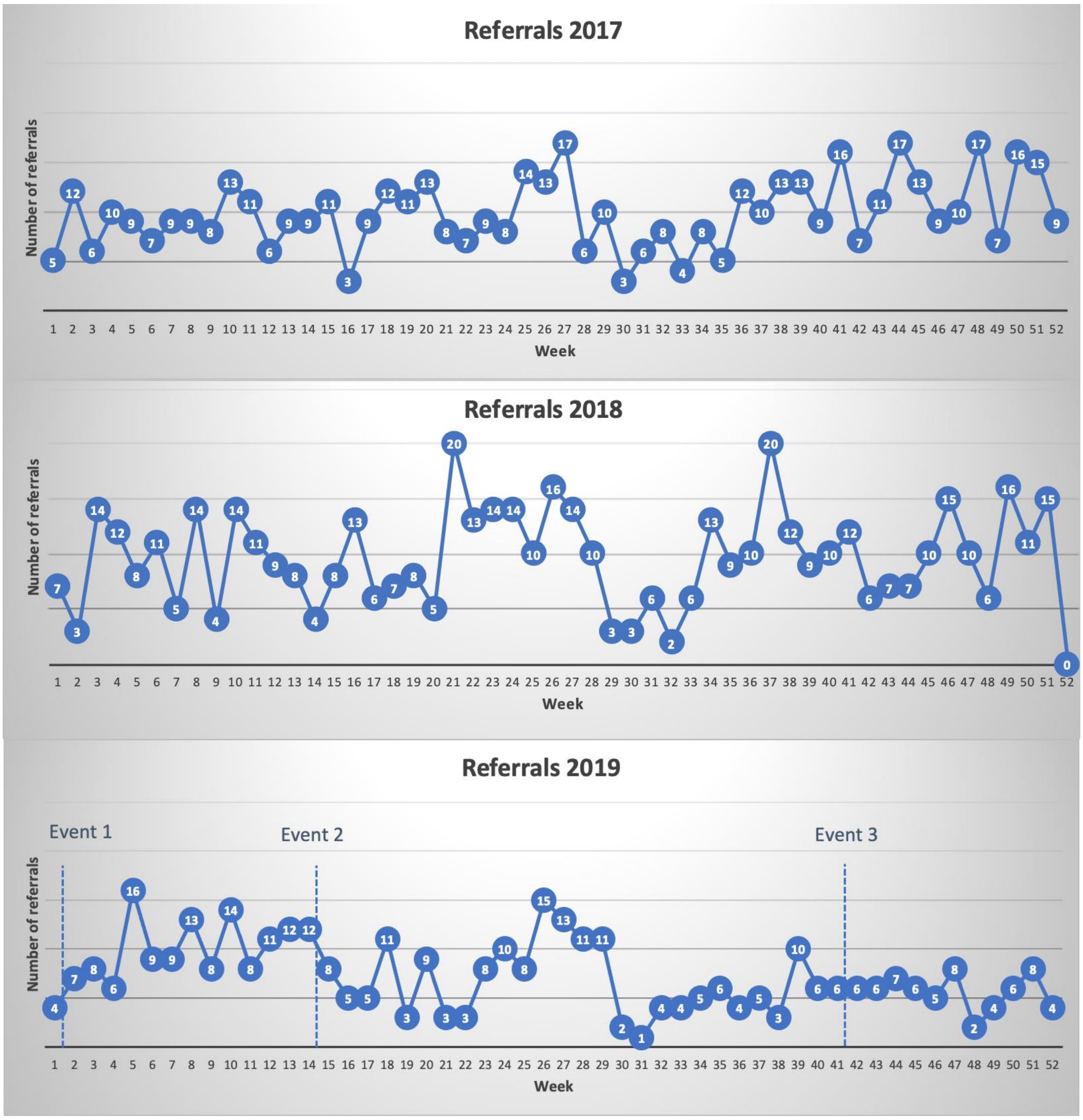

[a] Weekly referrals (n=1390) were provided by four of six Gender Identity Clinics.

© 2022 Indremo M et al. *JAMA Network Open.*
